# Risk assessment of trace metals in *Solanum lycopersicum* L. (tomato) grown under wastewater irrigation conditions

**DOI:** 10.1007/s11356-023-25157-8

**Published:** 2023-01-16

**Authors:** Dalia Abd El-Azeem Ahmed, Dalia Fahmy Slima, Hatim M. Al-Yasi, Loutfy M. Hassan, Tarek M. Galal

**Affiliations:** 1 Botany Department, Faculty of Science, Tanta University, Tanta, 31527 Egypt; 2 Botany and Microbiology Department, Faculty of Science, Menoufia University, Menoufia, Egypt; 3 Present Address: Biology Department, Faculty of Science, Taif University, P.O. Box 11099, Taif, 21944 Saudi Arabia; 4 Botany and Microbiology Department, Faculty of Science, Helwan University, Cairo, 11790 Egypt

**Keywords:** Heavy metals, Translocation and bioaccumulation factors, Daily intake of metals, Hazard quotient

## Abstract

Heavy metal contamination of food crop plants is viewed as a global issue. Heavy metals like cadmium (Cd), copper (Cu), lead (Pb), chromium (Cr), zinc (Zn), nickel (Ni), arsenic (As), cobalt (Co), and mercury (Hg) are poisonous. Depending on their concentration and capacity for bioaccumulation, they can provide a range of health risks.This research sought to investigate the effects of toxic metals (TMs) on the growth characteristics of produced tomatoes grown under wastewater irrigation. Additionally, it looked into the potential repercussions of both domestic and foreign individuals consuming this plant. In south Cairo, Egypt, two study locations were looked into: a control site in Abu Ragwan, which received water from tributaries of the Nile River, and a contaminated site in El-Shobak El-Sharky, which had raw industrial wastewater. The nutrients of soil and tomato plants (N, P, and K) decreased (*P* < 0.01), while TMs increased (*P* < 0.001) significantly as a result of using wastewater for irrigation. Except for Cu, all examined TM accumulating in tomato plants’ roots as opposed to shoots had a bioaccumulation factor (BF) > 1. However, the tomato plant’s shoot had solely undergone Pb and Ni translocation and storage, with a translocation factor (TF) > 1. A significant amount of Fe (5000.1 mg kg^−1^), Pb (360.7 mg kg^−1^), and Mn (356.3 mg kg^−1^) were present in the edible fruits. The ingestion of contaminated crops increases the daily intake rate of metals (DIR). The values of the high hazard quotient (HQ) were obtained (2073.8 and 2558.9 for Pb, 574.0 and 708.3 for Cd, and 41.1 and 50.7 for Fe for adults and children, respectively). Therefore, tomato plants grown in soils irrigated with untreated wastewater may offer a greater danger to human health, indicating that they should not be grown as a crop for human consumption.

## Introduction

The continuous and rapid expansion of agriculture, society, and the economy, as well as the preservation of human health, depend on the quality and safety of the soil environment (Chen et al. 2016; Liu et al. 2022). Because poisonous heavy metals are not biodegradable and accumulate in living tissues along the entire food chain, they are seen as a global problem that affects food crops (Adekunle et al. 2009). Their accumulation in agricultural soils is likewise thought to be a significant problem for humanity. Base rock, landfills for solid or liquid waste, mineral fertilisers, pesticides, agricultural inputs, industrial emissions, and urban pollutants are only a few of the sources of toxic metals in soil.. The majority of TMs are found in industrial settings and are major environmental polluters (Mitra et al. 2022). Therefore, TMs buildup in the soil results in agricultural soil pollution, which affects the quality and safety of food (Jolly et al. 2013; Huang et al. 2014).

One of the ways that poisonous heavy metals can enter human tissues and endanger human health is through food crops, especially fruits and vegetables (Siddiqui 2010). Because they are rich in fiber, vitamins, minerals, carbs, and proteins, fruits and vegetables are healthy for your health (Cherfi et al. 2014). Food crops play a significant role in our diet and may include a range of both essential and dangerous metals, depending on the characteristics of the growth medium utilized (Waqas et al. 2015). Heavy metals accumulate in both edible and non-edible parts of plants, causing a variety of problems for humans (Jarup 2003). The majority of a person’s intake of heavy metals, or about 90% of it, comes from eating contaminated vegetables, with the remaining 10% coming from skin contact and breathing in polluted dust (Khan et al. 2014). When used in moderation, heavy metals are beneficial to human health; nevertheless, when used in excess, they can cause serious diseases. Some fruits and vegetables include nutrients that are crucial for psychological and biochemical health, but they shouldn't be ingested in excess since they may result in a range of metabolic symptoms (Maleki and Zaras 2008; Fawad et al. 2017). The levels of these metals in particular media and the period of exposure determine the health hazards associated with TMs. Even at modest doses, prolonged and chronic exposure to TMs may have negative health effects (Mahalakshmi et al. 2012).

The cultivated tomato, *Solanum lycopersicum* L., is a member of the Solanaceae family. Due to its widespread use as a fundamental component in a variety of raw, cooked, and processed cuisines, it is the most planted vegetable in the world. Tomatoes rank among the most popular vegetables consumed worldwide. found in abundance in tomatoes, has been linked to a number of health advantages, including a decreased risk of cancer and heart disease. Phylloquinone (vitamin K1), potassium, folate, and vitamin C are also abundant in them (FAO 2021). Tomato pulp can also be used as a skin cleanser for oily skin, and sliced fruits can be used as a first-aid treatment for burns, scalds, and sunburns. Furthermore, the tomato fruit skin contains lycopene, which helps treat an enlarged prostate and related urinary issues as well as prevent heart attacks. Tomatoes are a unique crop in Egypt because they can be cultivated in all governorates and are available throughout the year. It is the vegetable with the largest total production capacity and cultivated area (FAO 2021). The volume of tomatoes produced in Egypt in 2019 was about 6.8 million metric tonnes (www.statista.com).

A comprehensive assessment of heavy metal contamination and subsequent risks for ecosystems and humans is necessary to develop management measures, reduce soil insecurity, and handle climate change (Kumar et al. 2019; Lian et al. 2022). Additionally, large quantities of Egyptian tomatoes are shipped to other nations throughout the world. Determining the health risk to people in this regard requires knowledge of the nutritional values and TMs concentrations in fruits and vegetables (Roba et al. 2016). As a result, this study explores the possibility of TMs spreading to tomato edible sections and the potential effects on both domestic and foreign customers.

## Material and methods

### Experiment

**Fig. 1:**
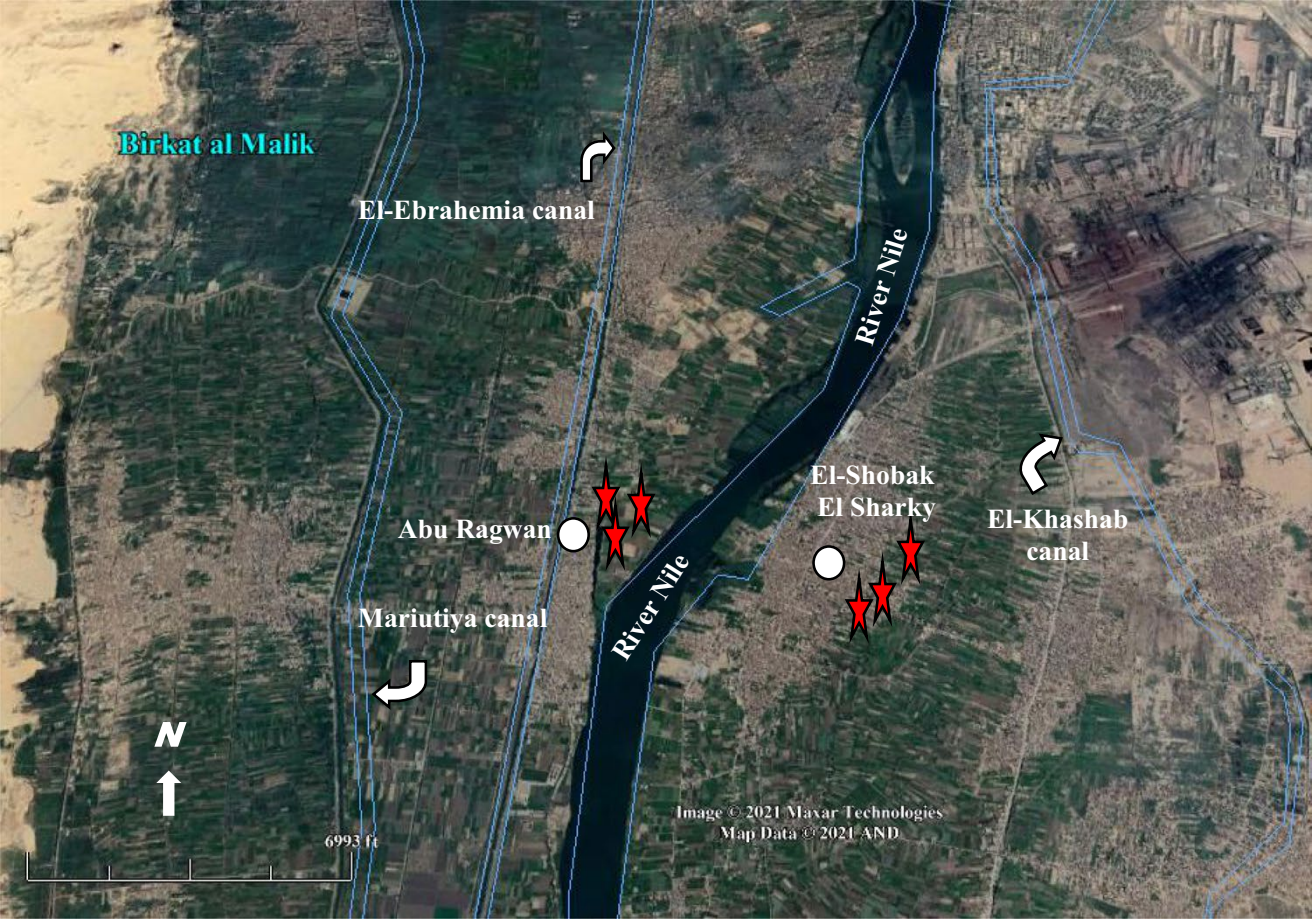
Location map of south Greater Cairo showing the study sites, 29° 44′ 56.54″ N; 31° 15′ 54.88″ E. Source: Google Earth on 7 June 2021

The sampling of tomato plants, as well as the irrigation water and soil samples, were collected from six farms (2 acres each) in south Greater Cairo, Egypt, during the summer of 2021. Three open farms are located at Abu Ragwan city (29° 47′ 6.57″ N and 31° 16′ 0.51″ E), receiving irrigation water from Nile River tributaries as control and the other three are located in the industrial area of El-Shobak El-Sharky city (29° 45′ 22.35″ N and 31° 17′ 36.20″ E) and receives irrigation water from untreated industrial effluents (Fig. 1). The farms’ owners permitted us to conduct the research on their property. The prevailing climate of the study area showed that the mean annual rainfall was 1.67–2.13 mm year^−1^, while the annual mean temperature was 21.08 °C, and the annual mean relative humidity was 52.68–56.08%.

#### Soil and water sampling and analysis

Three combined soil samples from each farm were collected at a depth of 0–50 cm, air-dried, and sieved through a 2 mm sieve to remove gravel and other debris. A soil water extract was prepared (1:5 *w/v*) for determining soil pH using a pH meter (Model 3520 JENWAY) according to Brower and Zar (1984) and electrical conductivity (EC) as an indicator of soil salinity using a conductivity meter (Model 4510 JENWAY) according to Roweel (1994). For soil nutrient (N, P, and K) and heavy metal (Pb, Cd, Cr, Cu, Ni, Fe, Mn, Zn, and Co) determination, the soil was digested using the acid digestion method (1 g soil sample was digested in 20 mL tri-acid mixture of HNO_3_/H_2_SO_4_/HClO_4_ (5:1:1, v/v/v) for 8 h at 80 °C, digestion was continued until the solution became clear, then the transparent digests were filtered using a 0.45 m pore size cellulose nitrate membrane filter paper (Millipore) and diluted up to 50 mL with distilled water then stored for analysis) according to Wade et al. (1993). The Kjeldahl method was used to determine total soluble nitrogen (N) according to Piper (1947), and the molybdenum blue method for determination of P content in soil samples using a spectrophotometer (V-760 UV–Visible spectrophotometer) at 660 nm for N and 700 nm for P. Simultaneously, the potassium (K) content was determined using a Flame Photometer (FP910-5 SHENZHEN). All previous procedures are listed in Allen (1989). The concentrations of the heavy metals investigated were determined using an Atomic Absorption Spectrophotometer (a Perkin-Elmer 3100) following APHA (1999).

In addition, three composite water samples were collected in plastic bottles from effluent received from untreated industrial wastewater and the river Nile. The pH/electric conductivity meter (914 pH/Conductometer-Metrohm AG) was used to measure the EC and pH of the water samples. The water samples were then directly acidified with nitric acid (1 ml HNO_3_/l) to determine the concentrations of Pb, Cd, Cr, Cu, Ni, Fe, Mn, Zn, and Co (APHA 1999). The relative oxygen requirements of wastewater were determined by measuring the chemical and biological oxygen demand (COD and BOD, respectively); COD was determined using titrimetric analysis (Pitwell 1983), while BOD was determined using the 5-day biochemical oxygen demand method (BOD5) (Delzer and Mckenzie 2003).

#### Plant sampling and growth measurements

Tomato plants were collected from the six farms under investigation. Ten quadrats (each 1 m^2^) were randomly selected at each farm for collecting tomato plants at the end of the growing season. Some growth parameters (number of individuals per m^2^, stem and root length, number of leaves per individual) of the harvested plants were measured. Fresh and dry weights of leaves and stems per m^2^, fresh and dry biomass, and productivity (t acre^−1^) were calculated using the collected samples divided into root, shoot, and fruits (edible part). According to Allen et al. (1986), dry weight for leaves and stems was determined by keeping samples at 40 °C for 3 days.

#### Heavy metals and plant nutrition

To measure the quantities of different harmful heavy metals (Pb, Cd, Cr, Cu, Ni, Fe, Mn, Zn, and Co), as well as plant nutrients (N, P, and K), proteins, and carbohydrates, the plant samples were divided into roots, shoots, and fruits before being air-dried and ground using an electric mill. One gram of each plant sample (control and contaminated) was digested using the acid-mixture digestion technique (Lu 2000). Ten milliliters of concentrated HNO_3_ (69%) and 0.5 mL of hydrofluoric acid (40%) were added to 1 g of dry plant sample in a closed Teflon vessel, which was then heated to 130 °C for 24 h. Until the mixture was clear, the digesting procedure was repeated. The samples were digested before being filtered through cellulose nitrate membrane filter paper at a 0.45 m thickness (Millipore). The volume was diluted with distilled water to 50 mL. An atomic absorption spectrophotometer was used to assess the content of heavy metals in plant samples (DW-AA320N), and nutrients (N, P, and K) were determined as mentioned in the “Soil and water sampling and analysis” section. Using a spectrophotometer (V-760 UV–Visible spectrophotometer) and the Bio-Rad protein assay (Lowry et al. 1951) and the anthrone-sulfuric acid techniques, the proportion of total soluble proteins and carbohydrates (Umbriet et al. 1959) was determined, respectively.

#### Plant pigments

According to Metzner et al. (1965), 2 g of fresh tomato leaves were extracted with approximately 20 mL of 50% acetone (v/v) in total darkness (light promotes chlorophyll degradation) and held at 4 °C overnight in order to identify leaf pigments (chlorophyll a, b, and carotenoids). The following pigments were measured: carotenoids = 4.2 E453—(0.0264 chl.a + 0.426 chl.b), Chl. a = 10.3 E663—0.918 E644, Chl. b = 19.7 E644—3.87 E663, and E stands for absorbance at a certain wavelength (nm).

### Data analysis

#### Soil–plant transfer of heavy metals

The following formula was used to determine the bioaccumulation factor (BF), which assesses a plant’s capacity to accumulate a particular metal in relation to soil concentration: C_root_ and C_soil_ are the corresponding heavy metal concentrations in the root and soil, respectively, and BF = C_root_/C_soil_. The translocation factor (TF), where C_shoot_ and C_root_ stand for the heavy metal concentrations in the plant’s shot and root, respectively, analyzes the relative translocation of metal from the plant’s root to the shoot (Ahmed et al. 2022).

#### Health risk assessment

The daily intake rate (DIR) of trace metals from tomato fruits was calculated using the following equation (Sharma et al. 2009): DIR = C_*heavy metal*_ × C_*factor*_ × D_*intake*_/BW, where C_*heavy metal*_ is the average heavy metal concentration in the edible parts (fruits) of tomato (mg kg^−1^); C_*factor*_ is a factor of 0.085 was used to convert the fresh to dry weight of these green fruits (Rattan et al. 2005); D_*intake*_ is the daily intake of tomato (0.345 and 0.232 kg person^−1^ day^−1^ FW) for adults and children, respectively; and BW is the average body weight (60.0 and 32.7 kg) for adults and children, respectively. The hazard quotient (HQ) for the consumers through the consumption of contaminated tomatoes was assessed as (USEPA 2013): HQ = DIR/R*f*D where R*f*D is the reference dose of heavy metals. The values of R*f*D for Pb, Cd, Cr, Cu, Ni, Fe, Mn, Zn, and Co were used as 0.001 (USEPA 2013) for Pb and Cd, 1.500 (USEPA 2013) for Cr, 0.040 (WHO 2013) for Cu, 0.020 (USEPA 2010) for Ni, 0.700 (WHO 2013) for Fe, 0.014(WHO 2013) for Mn, 0.300 (WHO 2013) for Zn and 0.043 mg kg^−1^ BW day^−1^ (USEPA 2013) for Co. The values of HQ < 1 are considered no risk, but if the values are > 1, they are supposed to be a high risk of TMs with long-term health hazard effects (Singh and Kumar 2017).

#### Statistical analysis

The differences in the analyzed variables in the soil, water, and plant at the studied sites were examined using the paired-sample *t*-test. Additionally, one-way analysis of variance (ANOVA) was utilized with SPSS software (version 23) to examine the data for normality and homogeneity of variance in addition to determining the significance of heavy metal changes among various plant organs (SPSS 2006).

## Results

### Soil and water characters

Irrigation with untreated industrial wastewater harmed soil characteristics studied (Table 1). All investigated characters were significantly increased (*P* < 0.05, *P* < 0.01, and *P* < 0.001) in contaminated farm soil. The control farm had an alkaline pH (pH = 8.25), a low EC (1.98 µs cm^−1^) and very low concentrations of the studied heavy metals. While the contaminated farm was slightly neutral pH (pH = 6.80), salinized (EC = 6.45 µs cm^−1^) and contained high levels of heavy metals (e.g., Fe = 194.33, Mn = 95.13, Zn = 91.67, and Cu = 24.37 mg kg^−1^). Furthermore, the PLI revealed that the soil of contaminated farms contained a high concentration of heavy metals; Pb had the highest PLI value (183.3), followed by Zn (39.0) and Cr (30.4).

**Table 1:** Soil characteristics (mean ± SD) and pollution load index (PLI) of tomato crops watered with untreated wastewater (contaminated farms) and Nile water (uncontaminated farms). Significant probability level: *P < 0.05, **P < 0.01, and ***P < 0.001

Soil characters		Farm		*t*-test	PLI
		Uncontaminated	Contaminated		
PH		8.25 ± 0.05	6.80 ± 0.02	18.6**	–
EC (µs cm^−1^)		1.98 ± 0.01	6.45 ± 0.06	22.6**	–
Total N (%)		2.95 ± 0.64	11.98 ± 3.25	114.3***	–
Total P (%)		4.45 ± 2.00	10.23 ± 2.27	28.6**	–
K	**mg kg**^**−1**^	25.78 ± 0.16	52.53 ± 3.44	132.4***	–
Pb		0.50 ± 0.01	91.67 ± 12.52	252.8***	**183.3**
Cd		0.03 ± 0.04	0.53 ± 0.02	64.4***	17.7
Cr		0.14 ± 0.00	4.26 ± 0.61	92.4***	30.4
Cu		3.05 ± 0.01	24.37 ± 2.67	98.6***	8.0
Ni		0.11 ± 0.01	2.25 ± 0.13	18.9**	20.5
Fe		13.70 ± 0.02	194.33 ± 2.08	172.3***	14.2
Mn		25.63 ± 0.03	95.13 ± 1.10	121.5***	3.7
Zn		2.35 ± 0.02	91.67 ± 3.79	256.6***	39.0
Co		0.11 ± 0.01	0.74 ± 0.01	8.3*	6.7

PLI = Cp/Cn, where Cp and Cn represent the toxic heavy metal concentrations in the soil of contaminated farm and soil of uncontaminated farm (control)

Analysis of irrigation water revealed that industrial wastewater was slightly alkaline (pH = 7.92), salinized (EC = 1664.33 µs cm^−1^), and had high BOD and COD values (673.33 and 1848.00 mg L^−1^, respectively) when compared to control. Furthermore, all heavy metals studied were significantly higher (*P* < 0.001 and *P* < 0.01) (Table 2). Furthermore, all chemicals (NO_3_, PO_4_, Cl, and SO_4_) were significantly higher (*P* < 0.01), particularly sulphates (758.00 mg L^−1^) and Cl (411.76 mg L^−1^).

**Table 2:** Irrigation water characteristics (mean ± SE). BOD and COD are the biological and chemical demand, respectively, TDS is total dissolved salts and Nd: not detected. *t-*value is provided. Significant probability level at: *P < 0.01 and **P < 0.001

Canal water variable		Farm		*t*-test
		Nile water	Untreated wastewater	
PH		7.59 ± 0.04	7.92 ± 0.23	8.9*
BOD	**mg l**^**−1**^	2.81 ± 0.04	*673.33* ± *6.66*	143.3**
COD		5.44 ± 0.07	*1848.00* ± *6.24*	97.8**
TDS		19.21 ± 0.30	1664.33 ± 5.51	65.7**
NO_3_		0.22 ± 0.03	3.14 ± 0.05	10.6*
PO_4_		0.09 ± 0.00	4.58 ± 0.03	2.3*
Cl		3.21 ± 0.03	411.67 ± 3.51	77.8*
SO_4_		3.96 ± 0.12	*758.00* ± *2.65*	53.7*
Pb		Nd	*0.49* ± *0.04*	183.3**
Cd		Nd	0.69 ± 0.03	17.7**
Cr		Nd	0.12 ± 0.01	30.4**
Cu		Nd	*2.43* ± *0.09*	8.0**
Ni		Nd	*0.55* ± *0.01*	20.5*
Fe		*0.01* ± *0.00*	*2.41* ± *0.06*	14.2**
Mn		0.04 ± 0.004	0.66 ± 0.04	3.7**
Zn		Nd	*2.01* ± *0.09*	39.0**
Co		Nd	*0.09* ± *0.00*	6.7*

### Growth parameters

**Fig. 2:**
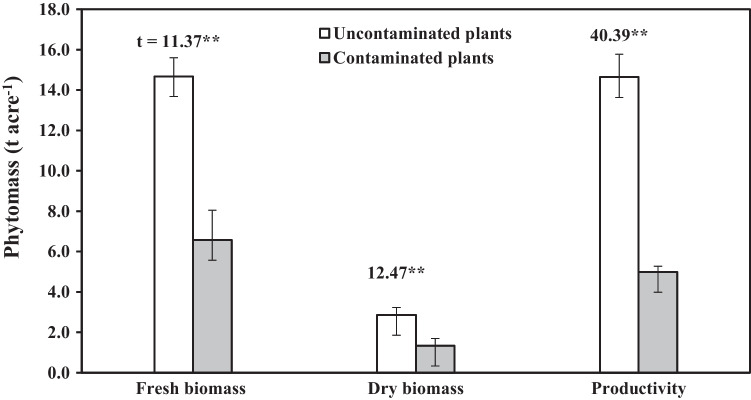
Fresh and dry biomass and production of tomato crop watered with untreated wastewater (contaminated farm) and Nile water (uncontaminated farm). Vertical bars are standard deviations. **p < 0.01

Plants watered with wastewater had a significant decrease (at *P* < 0.05, *P* < 0.01, and *P* < 0.001) in all studied growth parameters except the number of individuals, which remained constant (Table 3, Fig. 2). When compared to the control, stem length was reduced by 51.4%, root length by 46.3%, the number of leaves per individual by 51.6%, leaves fresh and dry weight by 57 and 53.4%, respectively, and stem fresh and dry weight by 55.2 and 53.2%, respectively. Furthermore, fresh and dry biomass was reduced by 55.2 and 53.1%, respectively, while productivity was reduced by 65.9%.

**Table 3:** Growth measurements (mean ± SD) of tomato plant (*N* = 60) watered with untreated wastewater (contaminated farms) and Nile water (uncontaminated farms). *P < 0.05, **P < 0.01, and ***P < 0.001

Plant parameter	Farm		*t*-test
	Uncontaminated	Contaminated	
Number of individuals m^−2^	1.00 ± 0.00	1.00 ± 0.00	0
Stem length (cm)	12.00 ± 1.0	5.83 ± 0.35	7.91*
Root length (cm)	13.33 ± 1.15	6.17 ± 0.25	13.36**
Number of leaves individual^−1^	73.00 ± 3.61	35.33 ± 3.51	16.14**
Leaves fresh weight (g m^−2^)	496.00 ± 18.33	213.33 ± 23.44	40.06***
Leaves dry weight (g m^−2^)	116.00 ± 8.0	54.00 ± 2.80	20.65**
Stem fresh weight (g m^−2^)	2792.00 ± 226.24	1249.33 ± 144.30	8.53*
Stem dry weight (g m^−2^)	545.47 ± 21.30	255.20 ± 37.19	9.94**

### Tomato plant analyses

#### Photosynthetic pigments

**Fig. 3:**
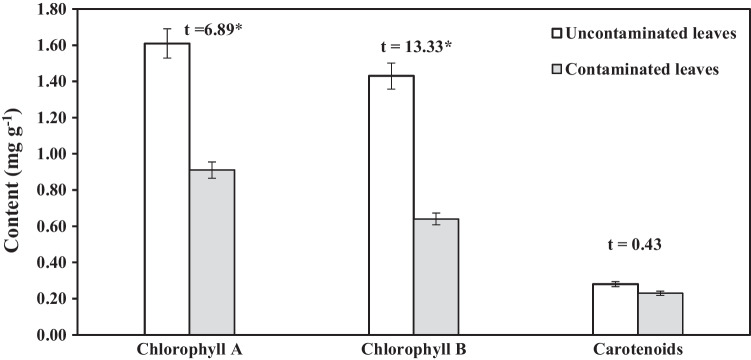
Photosynthetic pigments content of tomato leaves watered with untreated wastewater (contaminated farm) and Nile water (uncontaminated farm). Vertical bars are standard deviation. * *p* < 0.01

Photosynthetic pigments (chlorophyll a and b) in tomato leaves decline significantly (*P* 0.01) when the plants are watered with wastewater. Carotenoids had a non-significant decrement when compared to controls (Fig. 3). Carotenoids declined by 17.9%, while chlorophyll a and b decreased by 43.5 and 55.2%, respectively.

#### Nutrients and heavy metals

In shoot, the percentages of carbohydrates and proteins decrease from 18.95% and 14.96%, respectively, to 15.92% and 10% (Table 4). Furthermore, the percentages of N, P, and K in tomato shoots irrigated with wastewater decreased by 33.1%, 54.9%, and 18.8%, respectively (contaminated farms). While in roots, the percentages of carbohydrates and proteins decreased from 16.91% and 12.81%, respectively, to 14.3% and 7.35%, and N, P, and K decreased by 42.4%, 58.8%, and 61.6%, respectively. However, due to wastewater watering, the concentrations of all TMs studied (Pb, Cd, Cr, Cu, Ni, Fe, Mn, Zn, and Co) increased in the shoots, roots, and fruits (edible part) of tomatoes grown in contaminated farms (Tables 4 and 5). The difference percentage of accumulated TMs in tomato fruit (edible part) was Fe (98.9%), followed by Pb (93.8%), Mn (93.7%), and Zn (91.3%) (see Table 5).

**Table 4:** Nutrients and Heavy metals concentrations (mean ± SD) in the shoot and roots of tomato watered with untreated wastewater (contaminated farms) and Nile water (uncontaminated farms)

Nutrients	Uncontaminated farms		Contaminated farms	
	Shoot	Root	Shoot	Root
Organic (%)				
Carbohydrates	*18.95* ± *0.60a*	16.91 ± 3.14ab	15.92 ± 1.45ab	*14.30* ± *0.81b*
Proteins	*14.96* ± *1.62a*	12.81 ± 0.45b	10.00 ± 0.57c	*7.35* ± *1.14d*
Inorganic				
Total N (%)	*2.39* ± *0.26a*	2.05 ± 0.07b	1.60 ± 0.09c	*1.18* ± *0.18d*
Total P (%)	*2.57* ± *0.21a*	1.70 ± 0.20b	1.16 ± 0.05c	*0.70* ± *0.15d*
K (mg kg^−1^)	*25.50* ± *0.74a*	21.53 ± 1.07b	20.71 ± 0.17b	*8.26* ± *0.18c*
Heavy metals (mg kg^−1^)				
Pb	*41.67* ± *3.82d*	69.17 ± 3.82c	*420.00* ± *10a*	270.00 ± 13.23b
Cd	52.50 ± 15.21c	*41.67* ± *3.82c*	91.67 ± 7.64b	*130.00* ± *21.79a*
Cr	*0.27* ± *0.01c*	0.28 ± 0.03c	3.92 ± 0.15b	*4.42* ± *0.23a*
Cu	*0.29* ± *0.01c*	0.31 ± 0.01c	4.42 ± 0.08b	*4.90* ± *0.05a*
Ni	1.08 ± 0.14b	*0.75* ± *0.25b*	3.67 ± 0.29a	*3.68* ± *0.29a*
Fe	*668.33* ± *9.46d*	4008.33 ± 38.19c	5700.00 ± 56.35b	*7375.00* ± *90.14a*
Mn	*29.17* ± *3.82c*	66.67 ± 6.29b	295.00 ± 10.0a	*296.67* ± *16.07a*
Zn	*11.67* ± *3.82c*	42.50 ± 6.61b	53.33 ± 7.64b	*74.00* ± *11.53a*
Co	*0.42* ± *0.14d*	0.83 ± 0.14c	4.08 ± 0.13b	*4.47* ± *0.08a*

Means with the same letters in a row are not significant according to Duncan’s multiple range tests

**Table 5:** Heavy metals concentrations (mean ± SD) in the edible fruits of tomato watered with untreated wastewater (contaminated farms) and Nile water (uncontaminated farms)

Heavy metal (mg kg^−1^)	Farm		*t*-test	Difference (%)
	Uncontaminated	Contaminated		
Pb	22.25 ± 3.25	360.67 ± 40.37	56.99***	93.8
Cd	25.83 ± 3.44	99.83 ± 5.51	30.00***	74.1
Cr	0.29 ± 0.02	0.76 ± 0.02	52.92***	61.3
Cu	0.26 ± 0.01	0.63 ± 0.03	22.00**	58.7
Ni	0.61 ± 0.01	1.63 ± 0.06	43.86***	62.7
Fe	56.58 ± 6.74	5000.11 ± 242.10	878.37***	98.9
Mn	22.5 ± 2.50	356.33 ± 31.26	378.53***	93.7
Zn	5.17 ± 0.38	59.67 ± 4.16	22.69**	91.3
Co	0.98 ± 0.12	2.42 ± 0.12	20.81**	59.4

#### Soil–plant transfer of heavy metals

According to the findings in Table 6, tomato plants accumulate the majority of the TMs studied in their roots (BF values greater than one); Cd was the most accumulated metal (BF = 815), followed by Fe (BF = 165.26) and Pb (BF = 70.64). Tomato plants, on the other hand, accumulate only Pb (TF = 1.08) and Ni (TF = 1.27) in their shoots (TF values greater than 0ne), with little or no strategy for accumulating heavy metals in their fruits (edible part).

**Table 6:** Bioaccumulation (BF) and translocation (TF) factors of heavy metals in tomato plants watered with untreated wastewater (contaminated farms). Values > 1 are in bold

Heavy metal	BF	TF	
		Shoot	Fruit
Pb	**70.64**	**1.08**	0.83
Cd	**815.97**	0.98	0.69
Cr	**1.52**	0.94	0.61
Cu	0.15	0.95	0.48
Ni	**4.00**	**1.27**	0.66
Fe	**165.26**	0.47	0.35
Mn	**2.86**	0.72	0.77
Zn	**9.45**	0.50	0.46
Co	**6.79**	0.71	0.86

### DIR of metals and HQ

The present investigation showed that the DIR increased due to the consumption of wastewater irrigated crops grown in contaminated soil (Table 7). The DIR for the toxic heavy metals studied shows that the DIM for Pb (2.0738 and 2.5589), Fe (28.75 and 35.474), and Mn (2.0489 and 2.5281) are higher in adults and children who consume tomatoes grown in contaminated soil due to untreated wastewater irrigation, respectively.

**Table 7:** Daily intake rate (DIR) and hazard quotient (HQ) of heavy metals in tomato fruits watered with untreated wastewater (contaminated farms) and Nile water (uncontaminated farms). A: adult, C: children, and R_f_D: reference dose of trace metals. Values > 1 are in bold

Heavy metal	Contaminated farms				Uncontaminated farms				R_f_D	References
	DIR (mg day^−1^)		HQ		DIR (mg day^−1^)		HQ			
	A	C	A	C	A	C	A	C		
Pb	**2.0738**	**2.5589**	**2073.8333**	**2558.8583**	0.6525	0.4388	**652.4813**	**438.7700**	0.0010	(US-EPA 2013)
Cd	0.5740	0.7083	**574.0417**	**708.2977**	0.7576	0.5094	**757.5625**	**509.4333**	0.0010	(US-EPA 2013)
Cr	0.0044	0.0054	0.0029	0.0036	0.0086	0.0058	0.0057	0.0038	1.5000	(US-EPA 2013)
Cu	0.0036	0.0044	0.0898	0.1109	0.0076	0.0051	0.1894	0.1274	0.0400	(FAO/WHO 2013)
Ni	0.0094	0.0116	0.4696	0.5794	0.0179	0.0120	0.8932	0.6006	0.0200	(US-EPA 2010)
Fe	**28.7500**	**35.4740**	**41.0714**	**50.6772**	**1.6593**	**1.1158**	**2.3704**	**1.5940**	0.7000	(FAO/WHO 2013)
Mn	**2.0489**	**2.5281**	**146.3512**	**180.5796**	0.6598	0.4437	**47.1295**	**31.6929**	0.0140	(FAO/WHO 2013)
Zn	0.3431	0.4233	**1.1436**	**1.4111**	0.1515	0.1019	0.5050	0.3396	0.3000	(FAO/WHO 2013)
Co	0.0139	0.0171	0.3232	0.3987	0.0288	0.0194	0.6695	0.4502	0.0430	(US-EPA 2013)

For many studied TMs (Pb, Cd, Fe, Mn, and Zn), the HQ for consumers (adults and children) through the consumption of contaminated tomatoes causes a high risk in the long term (as HQ values more than one) (Table 7). For adults and children, the HQ values are 2073.8 and 2558.9 for Pb, 574.0 and 708.3 for Cd, 41.1 and 50.7 for Fe, 146.4 and 180.6 for Mn, and 1.1 and 1.4 for Zn.

## Discussion

### Soil and water characters

It is only possible to prevent heavy metal accumulation in crops’ edible components, such as fruits, by using untreated wastewater for irrigation. The physical and chemical properties of the soil are one factor that affects the uptake of heavy metals by vegetation, which is a complex phenomenon (Galal et al. 2021). When a farm was watered with untreated industrial wastewater, the soil’s pH declined while its salinity (EC) rose, indicating that the soil became more saline (EC = 6.45 S cm^−1^) and somewhat acidic (6.8). Low pH and high salinity increase the availability, mobility, and redistribution of heavy metals in different soil fractions (Ahmed et al. 2022). The current study found that irrigation with wastewater increased the levels of nutrients and heavy metals. According to Slima and Ahmed (2020), the increase in nutrient concentrations in polluted soil may be the result of absorption competition with accessible heavy metals, which became more mobile and available as salinity rose (Nzediegwu et al. 2020). High levels of Pb (PLI = 183.3) saturated the polluted soil. Pb was overloaded in soil because it was a relatively immobile heavy metal compared to others that were more mobile (Cu, Cd, and Co) (Ahmed and Slima 2018).

Additionally, the untreated wastewater utilised for irrigation had significantly high BOD (673.33 mg L^−1^) and COD (1848.00 mg L^−1^) values in comparison to the control, according to the current study. As a result, there is a rise in BOD and COD due to the high concentration of contaminants in the river, particularly organic matter. As a result, the water was no longer fit for drinking, irrigation, or other purposes. Additionally, untreated wastewater has high TM concentrations, which contaminates the soil and, as a result, the crops grown in areas that use it for irrigation (Gatta et al. 2015). Galal and Shehata (2015), Farahat et al. (2017), Slima and Ahmed (2020), and Ahmed et al. (2022) reported the same results.

### Growth parameters

Similar harmful effects of heavy metals on plants, both necessary and non-essential, include growth inhibition, low biomass output, photosynthesis, and nutrient assimilation, all of which result in plant death (Singh et al. 2016). With the exception of population, which stayed steady, all growth metrics of tomatoes watered with untreated wastewater in the current study were decreased. This result is in line with those of Mami et al. (2011), who found that tomato plants watered with water having a high concentration of trace metals, particularly Fe, Pb, and Cu, experienced a decline in growth metrics. Marwari and Khan (2012) reported that root and shoot length, root and shoot dry weight, and total dry weight were reduced to 50.55, 52.06, 69.93, 72.42, and 72.10%, respectively, in tomato plants irrigated with textile wastewater. According to Asati et al. (2016), heavy metal-contaminated soil caused rice plants to grow much shorter and produce fewer tillers and panicles. In addition to the Cd pollution in the soil (Cd as low as 5 mg/L), which inhibits the growth of wheat plants’ shoots and roots.

### Tomato plant analyses

#### Photosynthetic pigments

The amount of photosynthetic pigments significantly decreased when untreated wastewater was used for irrigation. Marwari and Khan (2012) found that chlorophyll content was severely affected by the increase in heavy metal concentration and the total chlorophyll content showed a reduction of 72.44% in tomato plants irrigated with textile wastewater. Heavy metals’ impact on CO_2_ assimilation, either by reducing the activity of the carboxylase enzyme or by reducing the ATP and reductant pool, maybe the cause of the decline in photosynthetic pigments (Singh et al. 2016). The same finding was made in *Pisum sativum*, *Abelmoschus esculentus*, and Japanese mustard spinach (Galal et al. 2021; Ahmed et al. 2022). (Abul Kashem and Kawai 2007). Mn, Cd, Cu, Ni, and Zn decrease the chlorophyll content (Maleva et al. 2012). Additionally, Dong et al. (2005) found that Cd stress had a detrimental effect on the rate of photosynthetic activity.

#### Nutrients and heavy metals

The concentration of N, P, and K in various plant organs significantly decreased when untreated wastewater was used to irrigate tomato plants (root and shoot). Begum et al. (2011) reported that a significant quantity of Pb metal was found in rice plant irrigation water as a result of water contamination from textile effluents, which resulted in a decrease in plant nutrients. Pb metal has the greatest percentage of any metal in the plant shoot in the current investigation (420 mg/kg). According to Osawa and Tajuke (1990), Cu had an adverse influence on N and decreased its content. In the current study, it was shown that tomato plants irrigated with untreated wastewater had lower levels of photosynthetic pigments, which in turn resulted in lower levels of carbs and proteins. This result corroborated those of Galal (2016) on *Cucurbita pepo*, Ahmed and Slima (2018) on *Corchorus olitorius*, Slima and Ahmed (2020) on *Pisum sativum*, and Ahmed et al. (2022) on *Abelmoschus esculentus*. According to Palma et al. (2002), under stress, the protease enzyme’s activity rises, leading to increased protein degradation and, eventually, a decrease in the amount of protein in plant tissues. Marwari and Khan (2012) reported that carbohydrate, protein and nitrogen content had a reduction of 46.83, 71.65, and 71.65% respectively in tomato plants irrigated with textile wastewater.

The concentration of the investigated heavy metals significantly increased when untreated wastewater was used to water plants (Table 4). With the exception of lead (420 mg/kg), all heavy metals investigated for uptake and distribution are detected in higher amounts in the tomato plant's root than in its shoot. This diminishing pattern in plant parts from the root to the shoot, with the exception of Pb, points to inadequate translocation of the examined heavy metals. The wastewater-irrigated fruit has a higher concentration of all tested heavy metals than the control (Table 5) and the highest concentration of heavy metals in contaminated tomato fruit is Fe with 98.9% difference from the uncontaminated one. Salem et al. (2016) found that Fe accumulated in all tomato parts including fruit. And With regard to tomato, peanut, and a number of plant species, the current study concurred with (Frederick and Ching 2014), (Ching, 2008), and (Peng et al. 2005). All of these data lend credence to the concept that some plant species' roots can gather metal contaminants and keep them from getting to the aerial sections of the plant. A similar finding was made on the okra plant by Ahmed et al. in 2022, suggesting that it is a hyperaccumulator for all heavy metals investigated. This outcome might be explained by the ability of certain ions to pass through physiological barriers such as the endodermis in tissues and the plasmalemma in cells (Seregin et al. 2003) or compartmentalization in cell vacuoles (Cheng 2008). While Gupta et al. (2008) found that although tomato plants show some phenotypic changes, the survival of tomato plants as well as least accumulation of metals in fruit reveals their tolerance to heavy metals and also it may be suggested that this plant can be grown successfully in the heavy metal contaminated soil.

#### Soil–plant transfer of heavy metals

Calculating BF and TF, all of which rely on the type of plant, confirms the mobility of trace metals from soil to plant root and subsequently from root to shoot (Eltaher et al. 2019). The plant can store the metal in its root when the BF value is higher than one, but it can translocate and store it in its shoot when the TF value is higher than one (Ahmed et al. 2022). The results in Table 6 show that tomato plants store the majority of the examined heavy metals in their roots (BF values greater than one). In contrast, Gupta et al. (2008) reported that the transfer factor of heavy metals from soil to tomato plants shows average value of less than 1, suggesting less uptake of heavy metals from soil. TF values greater than one, however, only Pb (TF = 1.08) and Ni (TF = 1.27) are translocated to their shoots, and these processes are either ineffective or nonexistent. Nitu et al. (2019) reported that there is a tendency to increase the final heavy metal content noticed in the tomato fruit and a decrease in the transfer coefficient of heavy metal in the fruit at the end of the vegetation period, and despite the high concentration of Zn, Cu, and Pb in tomato fruits, they still show a low risk for human consumption; due to the weak bioaccumulation transfer factors of heavy metals from soil. Murtć et al. (2018) show that the low accumulation of heavy metals in tomato fruits is the result of a synergy of different plant defense mechanisms that limit or reduce heavy metal transport from root to fruits.

### DIR of metals and HQ

Numerous potential routes for human exposure to pollutants must be investigated in order to determine their hazard quotient (health risk), with the food chain being the most significant (Galal 2016). The DIR for the analyzed hazardous heavy metals (Pb, Fe, and Mn) in the reference plant and contaminated plants exceeded 1 in both adults and children. Since tomato plants were grown in polluted soil due to untreated wastewater irrigation rather than controlled soils, the tested metals (apart from Pb, Fe, and Mn) posed essentially no risk to both adults and children who consumed them (WHO 1996). Additionally, according to Horiguchi et al. (2006), some of the ingested toxic heavy metals are expelled, while the remaining are stored in body tissues that have an impact on human health, so the eaten dose of toxic heavy metals is not equivalent to the absorbed pollutant dose in reality. The HQ is frequently employed to evaluate the risk of harmful substances in foods (Asgari and Cornelis 2015). The FAO/WHO (2013) and US-EPA (2013) both deem HQ values larger than one to be hazardous to human health. In the current study, the HQ for consumers (adults and children) from eating contaminated tomatoes offers a high risk in the long term (as HQ values surpass one) to local Egyptians for many of the heavy metals evaluated (Pb, Cd, Fe, Mn, and Zn) (Table 7). Due to the synergistic effects of metal combinations like Cd and Zn, there may be a risk (Symeonidis and Karataglis 1992). Furthermore, Pb and Cu work together, while Zn counteracts the toxicity of other metals like Cd, Ni, Mn, and Co, according to Chu and Chow (2002).

## Conclusion

The biomass and productivity of tomato plants growing in soil irrigated with untreated industrial wastewater are greatly reduced. Also, irrigation with wastewater reduced the amount of photosynthetic pigments in tomato leaves. The majority of the investigated heavy metals are deposited in the roots (BF > 1) of tomato plants and Cd was the most accumulated metal, followed by Fe and Pb (BF = 815.97, 165.26 and 70.64, respectively). It is worth to note that the edible parts (fruits) did not accumulate heavy metals except Pb and Ni with TF > 1. The investigated metals (except Pb, Fe, and Mn) offered little risk to both adults and children. However, Pb, Cd, Fe, and Mn in the polluted and Pb, Cd, Fe, Mn, and Zn in the unpolluted plants had HQ values greater than 1. Therefore, customers will be at danger for health concerns because local inhabitants consume a lot of tomatoes. Growing tomatos as a cash crop in contaminated soil for consumption by the public is not recommended due to the great danger they offer to human health.

## Data Availability

Please contact the authors for data requests.
